# Aβ_43_ is neurotoxic and primes aggregation of Aβ_40_ in vivo

**DOI:** 10.1007/s00401-015-1419-y

**Published:** 2015-04-11

**Authors:** Sylvie Burnouf, Marianna Karina Gorsky, Jacqueline Dols, Sebastian Grönke, Linda Partridge

**Affiliations:** Max Planck Institute for Biology of Ageing, Joseph-Stelzmann-Strasse 9b, 50931 Cologne, Germany; CECAD Cologne Excellence Cluster on Cellular Stress Responses in Aging Associated Diseases, 50931 Cologne, Germany; Department of Genetics, Evolution and Environment, Institute of Healthy Ageing, University College London, London, UK

**Keywords:** Alzheimer’s disease, Amyloid-β, *Drosophila* models, Neurodegeneration, Neurotoxicity

## Abstract

The involvement of Amyloid-β (Aβ) in the pathogenesis of Alzheimer’s disease (AD) is well established. However, it is becoming clear that the amyloid load in AD brains consists of a heterogeneous mixture of Aβ peptides, implying that a thorough understanding of their respective role and toxicity is crucial for the development of efficient treatments. Besides the well-studied Aβ_40_ and Aβ_42_ species, recent data have raised the possibility that Aβ_43_ peptides might be instrumental in AD pathogenesis, because they are frequently observed in both dense and diffuse amyloid plaques from human AD brains and are highly amyloidogenic in vitro. However, whether Aβ_43_ is toxic in vivo is currently unclear. Using *Drosophila* transgenic models of amyloid pathology, we show that Aβ_43_ peptides are mainly insoluble and highly toxic in vivo, leading to the progressive loss of photoreceptor neurons, altered locomotion and decreased lifespan when expressed in the adult fly nervous system. In addition, we demonstrate that Aβ_43_ species are able to trigger the aggregation of the typically soluble and non-toxic Aβ_40_, leading to synergistic toxic effects on fly lifespan and climbing ability, further suggesting that Aβ_43_ peptides could act as a nucleating factor in AD brains. Altogether, our study demonstrates high pathogenicity of Aβ_43_ species in vivo and supports the idea that Aβ_43_ contributes to the pathological events leading to neurodegeneration in AD.

**Electronic supplementary material:**

The online version of this article (doi:10.1007/s00401-015-1419-y) contains supplementary material, which is available to authorized users.

## Introduction

Alzheimer’s disease (AD) is a devastating neurodegenerative disorder characterized by the presence of two neuropathological hallmarks, namely the intraneuronal deposition of hyperphosphorylated Tau proteins into neurofibrillary tangles and accumulation of Aβ peptides both intracellularly and into extracellular amyloid plaques. Aβ peptides are produced following the sequential proteolytic cleavage of their precursor protein, APP, by secretases. The cleavage releasing the C-terminal part of Aβ can occur at different residues and hence produce peptides of different lengths, ranging from 37 to 49 amino acids [2], among which Aβ_40_ and Aβ_42_ are the most abundant [32]. Aβ_40_ species are soluble and abundantly produced in both healthy and AD brains. In contrast, Aβ_42_ levels are substantially increased in AD brains. Because of their high propensity to aggregate due to their two additional hydrophobic residues, Aβ_42_ peptides are the main constituents of amyloid deposits [36] and many studies have shown that they are highly pathogenic in the context of AD [15, 37].

Interestingly, recent studies have pointed to the potential of other Aβ species, and in particular of Aβ_43_, to be involved in AD pathogenesis. Indeed, Aβ_43_ is significantly increased in AD brains, deposits more frequently than Aβ_40_ and is found in the core of amyloid plaques [13, 17, 27, 30, 36]. Moreover, recent data suggest that Aβ_43_ is highly amyloidogenic in vitro [3, 4, 15, 29] and reduces the viability of cultured neuronal cells when applied in the culture medium [1, 23, 29]. In addition, higher cortical Aβ_43_ levels have been associated with increased amyloid load and impaired memory in the APP/PS1-R278I transgenic mouse model [29].

Importantly, in addition to its ability to self-aggregate in vitro to induce neurotoxicity, Aβ_43_ has been suggested to initiate the seeding of other Aβ peptides. Its addition to a mixture of Aβ peptides was shown to accelerate the formation of Thioflavin T-positive amyloid structures in vitro, in a more potent manner than did Aβ_42_ or Aβ_40_ [29]. In addition, Aβ_43_ was shown to deposit earlier than other Aβ species in the brain of mouse models of AD [38] and to be surrounded by other Aβ species in brains of AD patients [29], further suggesting its ability to nucleate and subsequently titrate other Aβ species.

However, a direct in vivo demonstration that Aβ_43_ self-aggregates, triggers neurotoxicity and exacerbates neurotoxicity from other Aβ species is so far lacking. The fruit fly *Drosophila* has proved an excellent in vivo model system for the analysis of both loss of function [10, 25] and toxic gain of function [5, 24] human neurodegenerative diseases. We have thus generated inducible transgenic *Drosophila* lines expressing human Aβ_43_, Aβ_42_ or Aβ_40_, using an attP/attB site-directed integration strategy to ensure both standard levels of mRNA expression and the best ratio of induced versus basal expression [20]. We observed that Aβ_43_ was highly insoluble in vivo and that it led to severe toxic effects, both when constitutively expressed in the compound eye of the fly, leading to eye roughness, and when specifically induced in the adult nervous system, as measured by a progressive loss of photoreceptor neurons, impaired locomotion and decreased lifespan. Interestingly, by combining transgenes encoding different Aβ isoforms we also found that, in presence of Aβ_43_, Aβ_40_ species were progressively shifted from the soluble to the insoluble protein fraction and that the overall Aβ insolubility was increased, leading to significant defects in climbing ability and survival. Altogether, our results demonstrate high pathogenicity of Aβ_43_ species in vivo and delineate their ability to trigger toxicity from the otherwise innocuous Aβ_40_.

## Materials and methods

### Generation of transgenic fly lines

**Fig. 1:**
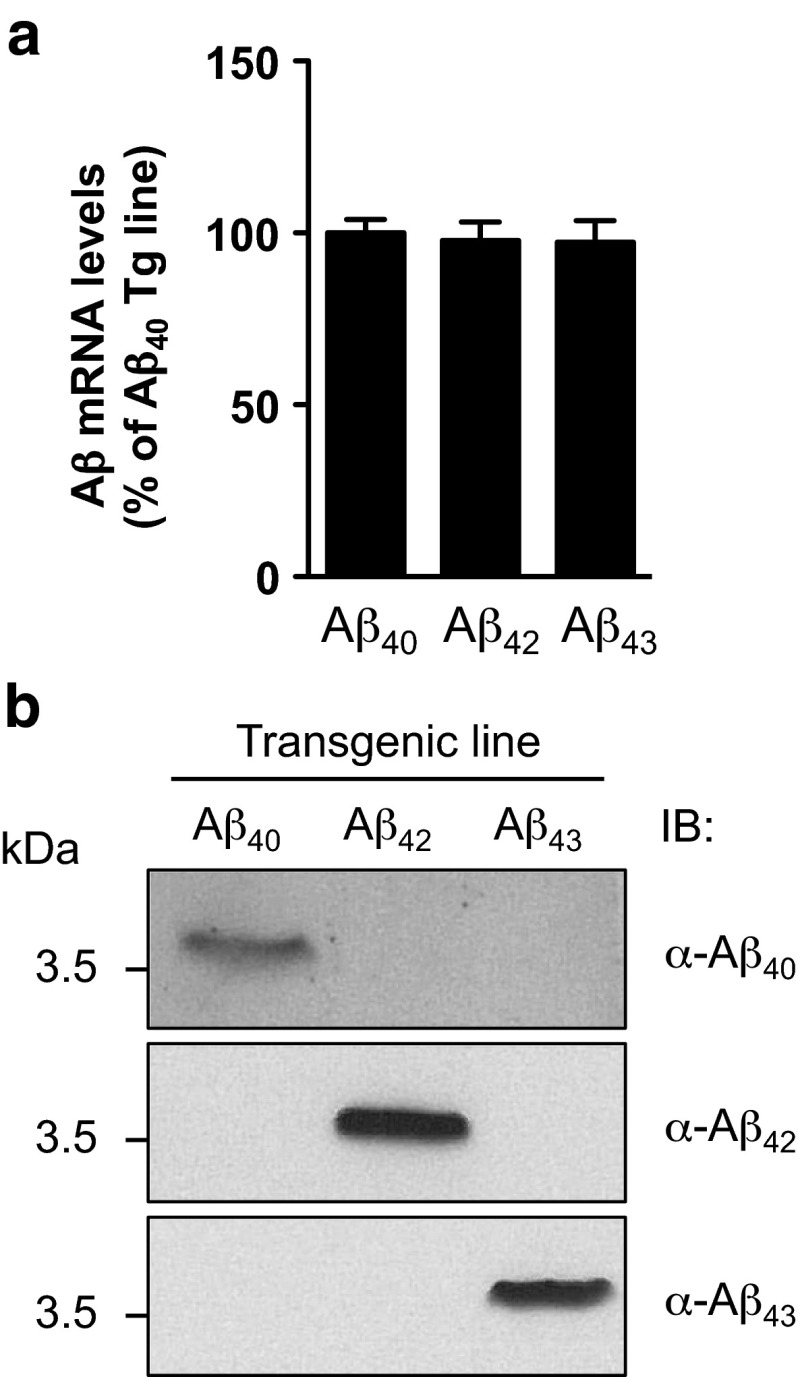
Expression of Aβ in fly heads. **a** qRT-PCR analysis of Aβ mRNA levels measured in head extracts of Aβ_40_, Aβ_42_ and Aβ_43_ transgenic lines (*p* > 0.05, one-way ANOVA) following expression in the fly nervous system using the neuron-specific elavGS driver. **b** Western blot analysis of head protein extracts from Aβ_40_, Aβ_42_ and Aβ_43_ transgenic fly lines (elavGS driver) probed with Aβ_40_-, Aβ_42_-, and Aβ_43_-specific antibodies, showing the high specificity of the transgenic lines

Human Aβ_1–40_, Aβ_1–42_ and Aβ_1–43_ bearing a secretion signal from the *Drosophila* necrotic gene [6] were synthesized by MWG Operon (Germany) using insect optimized codons and were subsequently cloned into the pUASTattB vector. An identical cDNA was used for all sequences that were common to all lines. Transgenic fly lines were generated using the φC31 and attP/attB targeted integration system and transgenes were inserted into the attP40 or attP2 landing-site loci to ensure both standard levels of mRNA expression and the best ratio of induced versus basal expression [20]. Transgenic fly lines were verified by sequencing of the corresponding genomic DNA and by western blotting (Fig. 1b). The gene-switch Elav-Gal4 inducible line (elavGS) was derived from the original elavGS 301.2 line [26] and obtained as a generous gift from Dr. H. Tricoire (CNRS, France). The GMR-Gal4 line was obtained from the Bloomington *Drosophila* Stock Center.

### Fly maintenance

All fly stocks were kept at 25 or 29 °C on a 12:12 h light:dark cycle at constant humidity and fed with standard sugar/yeast/agar (SYA) medium (15 g L^−1^ agar, 50 g L^−1^ sugar, 100 g L^−1^ yeast, 30 mL L^−1^ nipagin and 3 mL L^−1^ propionic acid). All lines were backcrossed into a white Dahomey (w^Dah^) wild-type outbred strain for at least ten generations prior to experiments. Adult-onset transgene expression was achieved using the inducible gene-switch UAS-Gal4 system and through addition of the activator RU486 (Mifepristone) to fly food at a final concentration of 200 µM. Non-induced controls were obtained by adding the vehicle (i.e. ethanol) to fly food. All experimental flies were kept at 25 °C throughout development and during the 48-h mating step following eclosion, after which females were sorted and transferred to 29 °C. Only flies used for the eye phenotype experiment were kept at 25 °C, since an eye phenotype can already be observed in the GMR-Gal4 driver control at 29 °C.

All investigated lines were homozygous for the Aβ transgene unless indicated by “1×Aβ”, meaning flies were heterozygous for the transgene.

### Lifespan analysis

For lifespan experiments, 200 once-mated females per group were allocated to vials at a density of 10 flies per vial and subsequently kept at 29 °C. Flies were transferred to new vials every 2–3 days and the number of dead flies was recorded. Lifespan results are expressed as the proportion of survivors ±95 % confidence interval.

### Climbing assay

Climbing assays were performed blindly using a countercurrent apparatus as previously described [9] using at least 3 replicates of 20 female flies per group. One hour prior to the measurement, flies were randomized and transferred to plastic tubes for acclimation. Flies were placed into the first chamber of the six-compartment climbing apparatus, tapped to the bottom and given 20 s to climb a distance of 15 cm, after which flies above this level were shifted to the second chamber. Both sets of flies were tapped again to the bottom and allowed to climb for another 20 s. This procedure was repeated for a total of 1 min and 40 s so that flies could climb into the six chambers, and the number of flies in each chamber was counted at the end of the experiment. The climbing index (CI) was calculated as previously described [9] and varied between 0 (all flies stayed in the first compartment) and 1 (all flies reached the last chamber).

### Eye phenotype

Eye images of 6-day-old female flies expressing Aβ under the control of the GMR-Gal4 driver at 25 °C were taken using a Leica M165 FC stereo microscope. At least 8 flies per genotype were investigated.

### Rhabdomere assay

We used the cornea neutralization technique [8] to visualize the rhabdomeres from the ommatidia of the fly compound eye. Briefly, dissected fly heads were mounted on a microscope slide using a drop of nail polish and further covered with oil. The number of ommatidia lacking rhabdomeres was counted using a Leica DMI4000B/DFC 340FX inverted microscope and a 40× oil immersion objective. At least 50 ommatidia per fly and 5 flies per genotype were examined.

### Total Aβ levels

20 to 25 female heads per biological replicate were homogenized in 100 µL of 70 % formic acid using a disposable pellet mixer and a plastic Eppendorf pestle. Samples were centrifuged at 16,000*g* for 20 min at room temperature. The supernatant was collected and subsequently evaporated using a SpeedVac. The dry pellet was resuspended in 100 µL 2× LDS containing reducing agent (Invitrogen) and homogenized by sonication (10 pulses). Samples were then boiled at 100 °C for 10 min and 10 µL of each sample were used for western blotting to determine total Aβ levels.

### Fractionation of soluble and insoluble Aβ peptides

The procedure was based on previous reports [7] with some modifications. Briefly, 20 female heads per biological replicate were homogenized in 100 µL ice-cold RIPA buffer (Pierce) supplemented with SDS at a final concentration of 1 % and Complete mini without EDTA protease inhibitor (Roche) using a disposable pellet mixer and a plastic Eppendorf pestle. Samples were incubated on ice for 30 min and were then centrifuged at 100,000*g* for 1 h at 4 °C in an Optima XPN-100 ultracentrifuge (Beckman Coulter). The supernatant (“soluble fraction”) was collected and the pellet was homogenized in 100 µL of 70 % formic acid by pipetting, followed by 5-min incubation in a sonication bath. Samples were centrifuged again at 100,000*g* for 1 h at 4 °C, after which the supernatant was collected (“insoluble fraction”) and evaporated using a SpeedVac. The dry pellet was then resuspended in 100 µL 2× LDS containing reducing agent (Invitrogen) by pipetting followed by 5-min incubation in a sonication bath. Protein concentration of the soluble fraction was measured using the BCA protein assay kit (Pierce), and 30 µg of soluble proteins and equivalent volumes of insoluble proteins were used for western blotting. Results are represented as the proportion of insoluble Aβ species, i.e. insoluble Aβ/(soluble Aβ + insoluble Aβ) × 100. Results are expressed as mean ± sem.

### Sample preparation for soluble Aβ oligomers and dot blotting

The procedure was based on previous reports [21] with some modifications. Briefly, 20 female heads per biological replicate were homogenized in 100 µL ice-cold 1× PBS buffer supplemented with Complete mini without EDTA protease inhibitor (Roche) using a disposable pellet mixer and a plastic Eppendorf pestle. Samples were then ultra-centrifuged at 78,000*g* for 1 h at 4 °C. The supernatant, i.e. the PBS-soluble fraction was collected and protein concentration was measured using the BCA protein assay kit (Pierce). One microlitre per sample (corresponding to 1.5 µg of proteins) was spotted onto a 0.2 µm nitrocellulose membrane (GE Healthcare) and let to dry for 30 min. The membrane was then blocked in TBS-low tween buffer (containing 0.01 % Tween) with 10 % non-fat dry milk for 1 h at room temperature, incubated overnight at 4 °C with the A11 anti-oligomer antibody (1/1000, Invitrogen) and then for 1 h at room temperature with HRP-conjugated anti-rabbit antibody (1/10,000, Invitrogen). Detection was performed using ECL prime chemiluminescence kits (GE Healthcare) and Hyperfilms (GE Healthcare).

### Sample preparation for LDS/SDS-stable Aβ oligomers

20 female heads per biological replicate were homogenized in 100 µL 2× LDS containing reducing agent (Invitrogen) using a disposable pellet mixer and a plastic Eppendorf pestle. Sample were incubated on ice for 30 min and were then boiled at 100 °C for 10 min. 15 µL per sample were used for western blotting to evaluate LDS/SDS-stable Aβ oligomers.

### Western blotting

Protein samples were separated on 16.5 % Tris-Tricine Criterion gels (Biorad) and subsequently transferred to 0.2 µm nitrocellulose membranes (GE Healthcare). After a boiling step of 4 min in 1× PBS, membranes were blocked in TNT buffer (Tris–HCl 15 mM pH 8, NaCl 140 mM, 0.05 % Tween) with 5 % non-fat dry milk for 1 h at room temperature and incubated overnight at 4 °C with the following primary antibodies: anti-Aβ_1–16_ mAb (6E10, 1/5000, Covance), anti-Aβ_1–40_ mAb (9682, 1/500, Cell Signaling), anti-Aβ_1–42_ mAb (12F4, 1/500, Covance), anti-Aβ_1–43_ mAb (9C4, 1/500, Covance), anti-α-tubulin (11H10, 1/5000, Cell Signaling) and anti-β-actin (1/100,000, Abcam). HRP-conjugated anti-mouse or anti-rabbit antibodies (1/10,000, Invitrogen) were used for 1 h at room temperature and detection was performed using ECL or ECL prime chemiluminescence kits (GE Healthcare) and Hyperfilms (GE Healthcare). Bands were quantified using the ImageJ software (Scion Software) and results are expressed as mean ± sem.

### Immunofluorescence

After decapitation and removal of proboscis, heads of 20-day-old GMR-Gal4-driven Aβ flies were fixed for 3 h in 4 % paraformaldehyde in PBS and then incubated overnight in 25 % sucrose in PBS. Heads were frozen in Tissue-Tek O.C.T. (Sakura Finetek) and kept at −80 °C until use. Immunofluorescence was performed on 16 µm cryosections using an anti-Aβ_1–40_ mAb antibody (D8Q71, 1/200, Cell Signaling) followed by anti-rabbit Alexa-488 secondary antibody (1/250, Invitrogen), both being diluted in PBS with 0.1 % Triton and 5 % non-fat dry milk. An incubation step of 3 min in 70 % formic acid was included prior to blocking to unmask antigens. Stained sections were mounted using Vectashield with DAPI (Vector) and analysed with a Leica DMI4000B/DFC 340FX inverted microscope. Quantification of Aβ_40_ deposits was performed using ImageJ from at least 6 flies per genotype.

### RNA extraction and qRT-PCR

Total RNA was extracted from 25 female heads per replicate using a Trizol-Chloroform-based procedure (Invitrogen) and subsequently treated with DNAse I (Ambion). 300 ng of RNA were then subjected to cDNA synthesis using the SuperScript Vilo Mastermix (Invitrogen). Quantitative real-time PCR was performed using TaqMan primers (Applied Biosystems) in a 7900HT real-time PCR system (Applied Biosystems). RPL32 and actin5c were used as normalization controls and the relative expression of target genes was determined by the ΔΔ*C*_T_ method. Six independent biological replicates per group were analysed. Results are expressed as a percentage of the corresponding control transgenic line and are plotted as mean ± sem.

### Statistical analysis

For lifespan experiments, statistical differences were assessed using the log-rank test. Eye phenotypes were statistically evaluated using the Fisher’s exact test. Other results are expressed as mean ± sem and differences between mean values were determined using either Student’s *t* test, one-way ANOVA followed by Tukey’s post hoc test or two-way ANOVA followed by Tukey’s post hoc test, using Graphpad Prism software. *p* values <0.05 were considered significant.

## Results

We generated transgenic *Drosophila* lines expressing human Aβ_43_, Aβ_42_ or Aβ_40_, using a site-directed integration strategy to allow transgene insertion into the same genomic locus and therefore ensure equivalent levels of Aβ mRNA expression among the lines, as verified by qRT-PCR (*p* > 0.05, Fig. 1a). Specific expression of individual Aβ species was checked by western blot using C-terminal-specific, anti-Aβ antibodies (Fig. 1b), confirming that each transgenic line was expressing a single Aβ species.

**Fig. 2:**
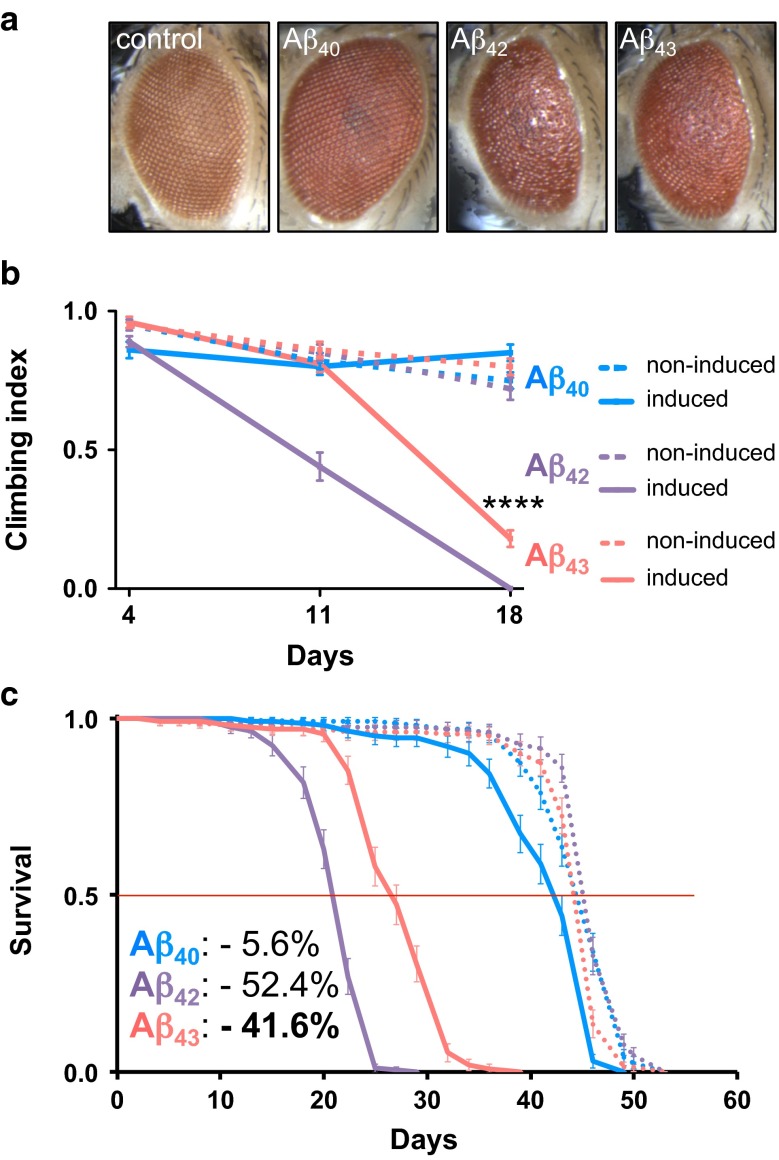
Aβ_43_ expression led to detrimental effects on fly eye appearance, climbing ability and survival. **a** Representative microphotographs of adult fly eyes upon Aβ over-expression using the constitutive, eye-specific GMR-Gal4 driver. Control eyes were obtained from the GMR-Gal4 driver line, which do not over-express any Aβ peptide. Climbing ability (**b**) and survival curves (**c**) of RU486-induced (*plain curves*) and non-induced (*dotted curves*) Aβ_40_ (*blue*), Aβ_42_ (*purple*) and Aβ_43_ (*red*) transgenic flies. The *inset* shows the percentage of median lifespan reduction versus non-induced control. ****p < 0.0001 versus non-induced controls, two-way ANOVA

We first examined the effect of human Aβ homozygous over-expression in *Drosophila* eyes, using the constitutive, eye-specific GMR-Gal4 driver. While the eyes of Aβ_40_-expressing flies were not affected and appeared similar to those of the driver line control, Aβ_43_ expression led to ommatidial disorganization and eye roughening, indicating that Aβ_43_ was able to induce toxicity in vivo (Fig. 2a). Quantification of the observed rough eye phenotypes among the lines confirmed a significant toxic effect of Aβ_43_ expression (**p < 0.01, Aβ_43_ vs. Aβ_40_ and Aβ_43_ vs. GMR driver control, Fisher’s exact test, supplementary Fig. 1), although this effect was significantly milder than the one induced by Aβ_42_ (#p < 0.05 and *p < 0.05, Aβ_43_ vs. Aβ_42_, Fisher’s exact test, supplementary Fig. 1). We then used the pan-neuronal and RU486-inducible elav-GeneSwitch-Gal4 (elavGS) driver [28] to investigate the effects on fly climbing ability and survival of the expression of human Aβ specifically in adult neurons. As expected from previous reports [7, 33], adult-restricted, neuronal expression of Aβ_42_ led to severe toxic effects, both on climbing ability (*p* < 0.0001 vs. non-induced controls after 11 and 18 days of induction, two-way ANOVA, Fig. 2b) and on fly survival (median lifespan: −52.4 % of non-induced controls, *p* < 0.0001, log-rank test, Fig. 2c), while induction of Aβ_40_ produced no overt defects in climbing ability (*p* > 0.05 vs. non-induced controls at all investigated ages, two-way ANOVA, Fig. 2b) or survival (median lifespan: −5.6 % of non-induced controls, Fig. 2c), similar to what we observed for the driver line control (climbing ability: *p* > 0.05 at all investigated ages using two-way ANOVA, supplementary Fig. 2a; median lifespan: −6.3 % of non-RU486-fed controls, supplementary Fig. 2b). In line with the toxicity measured following its specific over-expression in the fly eye, inducing Aβ_43_ expression in the adult nervous system severely impaired fly climbing performance (****p < 0.0001 vs. non-induced controls after 18 days of induction, two-way ANOVA, Fig. 2b), although the induced toxicity was significantly milder than following Aβ_42_ expression (*p* < 0.0001 and *p* < 0.001 after 11 and 18 days of induction, respectively, induced-Aβ_42_ vs. induced-Aβ_43_, two-way ANOVA, Fig. 2b). Survival showed a similar pattern, with neuronal expression of Aβ_43_ leading to a severe shortening of median lifespan, corresponding to −41.6 % of the non-induced controls (*p* < 0.0001, log-rank test, Fig. 2c). Our results therefore demonstrate strong toxicity from Aβ_43_ over-expression in adult neurons.

**Fig. 3:**
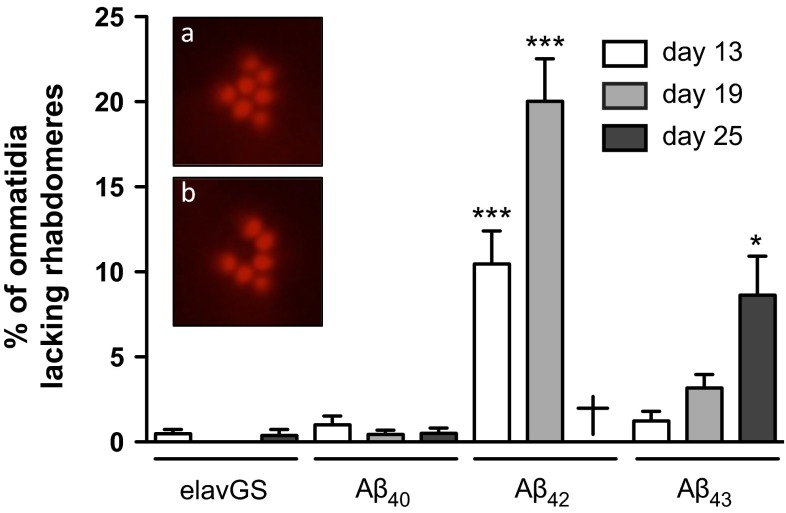
Aβ_43_ expression led to progressive neurodegeneration. The percentage of ommatidia lacking rhabdomeres was quantified from the RU486-induced Aβ_40_, Aβ_42_ and Aβ_43_ transgenic fly lines and elavGS driver control at three different time points [13 (*white*), 19 (*grey*) and 25 (*black*) days of induction, *p < 0.05 and ***p < 0.001 vs. age-matched elavGS driver control]. *Insets* show representative micrographs of the rhabdomeres from a normal ommatidium (**a**) and from an ommatidium lacking the central rhabdomere (**b**). All Aβ_42_-expressing flies were dead at day 25

To investigate whether neuronal expression of Aβ resulted in neuronal degeneration, we used the quantitative pseudopupil technique to analyse the loss of photoreceptor neurons from the fly compound eye [8, 14, 35]. Each ommatidium from the fly eye contains seven visible photoreceptor neurons that produce photosensitive structures called rhabdomeres. We evaluated the proportion of ommatidia showing a loss of rhabdomeres following Aβ expression in adult neurons (Fig. 3, the bottom inset showing an ommatidium lacking the central rhabdomere, to be compared with a normal ommatidium in Fig. 3, top inset). While expression of Aβ_40_ led to no significant loss of photoreceptor neurons as compared to the elavGS driver control (*p* > 0.05 vs. age-matched controls, at all investigated ages, Fig. 3), Aβ_42_-expressing flies displayed a progressive and pronounced neurodegeneration, the percentage of affected ommatidia reaching 10.48 ± 1.93 % at day 13 (***p < 0.001 vs. age-matched elavGS control, one-way ANOVA, Fig. 3) and 20.02 ± 2.52 % at day 19 (***p < 0.001 vs. age-matched elavGS control, one-way ANOVA, Fig. 3). Interestingly, we found that the expression of Aβ_43_ in adult neurons also triggered progressive loss of rhabdomeres with age (*p* < 0.01, one-way ANOVA), affecting 3.18 ± 0.79 and 8.64 ± 2.28 % of ommatidia at day 19 and 25, respectively, the latter being significantly higher than the effect observed in both elavGS controls and Aβ_40_-expressing flies (*p < 0.05 vs. 25-day-old elavGS driver controls and *p* < 0.01 vs. 25-day-old Aβ_40_ transgenic line, one-way ANOVA, Fig. 3), showing that Aβ_43_ peptides led to neurodegeneration in vivo. This neuronal loss was significantly milder than that observed following Aβ_42_ over-expression (*p* < 0.001 at day 13 and day 19, Aβ_43_ vs. Aβ_42_, one-way ANOVA; note that all Aβ_42_-expressing flies were dead at day 25). Altogether, our data demonstrate the ability of Aβ_43_ peptides to trigger toxicity and neurodegeneration in *Drosophila*, and suggest that the toxic effects are significantly milder than those induced by Aβ_42_ over-expression.

**Fig. 4:**
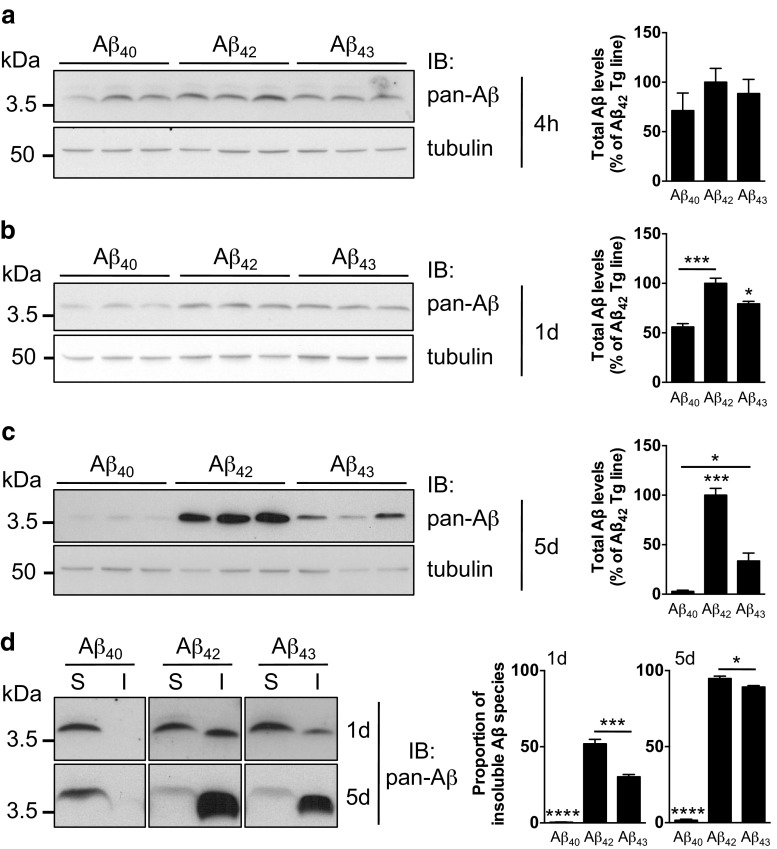
Total Aβ amounts and solubility following induction in the fly nervous system. Western blot analysis of total Aβ retrieved from fly head extracts following either 4 h (**a**), 1 day (**b**) or 5 days (**c**) of neuronal (elavGS-driven) expression of Aβ_40_, Aβ_42_ or Aβ_43_, using the pan-Aβ 6E10 antibody. Quantifications (*right panels*) were made using Tubulin for normalization and results are expressed relative to levels measured in the Aβ_42_ line (4 h induction: *p* > 0.05; 1 day induction: *p < 0.05, Aβ_43_ vs. Aβ_42_ and Aβ_43_ vs. Aβ_40_, ***p < 0.001, Aβ_42_ vs. Aβ_40_; 5 days induction: *p < 0.05, Aβ_40_ vs. Aβ_43_, ***p < 0.001, Aβ_42_ vs. Aβ_40_ and Aβ_42_ vs. Aβ_43_). **d** Fractionation of SDS-soluble (*S*) and SDS-insoluble/formic acid-soluble (*I*) Aβ species was performed from heads of elavGS-driven Aβ_40_, Aβ_42_ and Aβ_43_ transgenic flies following 1 or 5 days of induced expression and probed using the 6E10 pan-Aβ antibody. The quantifications of the proportion of insoluble Aβ species are shown on the *right panel* (***p < 0.001, Aβ_42_ vs. Aβ_43_ and ****p < 0.0001, Aβ_40_ vs. Aβ_42_ and Aβ_40_ vs. Aβ_43_ after 1 day of induced expression; *p < 0.05, Aβ_42_ vs. Aβ_43_; ***p < 0.001, Aβ_40_ vs. Aβ_42_ and Aβ_40_ vs. Aβ_43_ after 5 days of induced expression)

Since Aβ mRNA levels were comparable among the lines as measured by quantitative real-time PCR (Fig. 1a), we hypothesized that the different degrees of toxicity induced by over-expression of Aβ_40_, Aβ_42_ or Aβ_43_ could be explained by a differential stability of the Aβs at the peptide level. We therefore measured the total levels of Aβ peptides retrieved from fly heads following Aβ induction in the adult nervous system, using an antibody binding to the N-terminal region of Aβ that is common to all three peptides. First, we analysed total Aβ levels at a very early time point, following 4 h of RU486 induction, and we observed no significant difference between the lines (*p* > 0.05, one-way ANOVA, Fig. 4a). However, as early as 1 day following the beginning of transgene induction, we could already observe that total Aβ amounts significantly differed between the lines, with levels of Aβ_40_ and Aβ_43_ representing 55.92 ± 3.45 and 79.30 ± 2.55 % of Aβ_42_ levels, respectively (*p < 0.05 and ***p < 0.001, one-way ANOVA, Fig. 4b). Such differences intensified with time. Total Aβ peptide levels measured in the Aβ_40_-expressing line were extremely low compared to Aβ_42_ transgenics after 5 days of induction (2.79 ± 1.32 % relative to Aβ_42_ levels, ***p < 0.001, one-way ANOVA, Fig. 4c), in line with previous reports [7]. We also observed that total levels of Aβ were markedly lower in the Aβ_43_ than in the Aβ_42_ transgenics after 5 days of induction (33.60 ± 7.96 % relative to Aβ_42_ levels, ***p < 0.001, one-way ANOVA, Fig. 4c), but significantly higher than those retrieved from the heads of Aβ_40_ transgenic flies (*p < 0.05, one-way ANOVA, Fig. 4c).

These results suggest that these Aβ isoforms differentially accumulate in the fly nervous system. Such effects could be explained by a differential aggregation propensity and therefore protein stability of these three Aβ species. To tackle this question, we performed fractionation experiments to evaluate whether Aβ_40_, Aβ_42_ and Aβ_43_ would display an unequal propensity to form amyloid structures in vivo in the adult fly brain, as previously suggested by in vitro studies [4, 29]. Interestingly, fractionation of Aβ peptides according to their solubility in 1 % SDS revealed differences among the lines as early as 1 day following the start of induction in the fly nervous system. We confirmed previous reports from *Drosophila* [7, 12] of a high solubility of Aβ_40_ species (0.48 ± 0.10 and 1.61 ± 0.66 % of Aβ_40_ species being insoluble after 1 and 5 days of induction, respectively, Fig. 4d) while most of the Aβ_42_ was found in the insoluble fraction (51.92 ± 2.94 % at day 1 and 94.76 ± 1.61 % at day 5, ****p < 0.0001 vs. Aβ_40_, one-way ANOVA, Fig. 4d), suggesting that Aβ_42_ peptides form insoluble structures when expressed in adult fly neurons. Interestingly, 1 day after the beginning of induction, a significantly higher proportion of Aβ_43_ peptides than Aβ_40_ were found in an insoluble state (30.30 ± 1.50 %, ****p < 0.0001 vs. Aβ_40_, one-way ANOVA, Fig. 4d), although they were more soluble than Aβ_42_ peptides (***p < 0.001 vs. Aβ_42_, one-way ANOVA, Fig. 4d). The proportion of insoluble Aβ_43_ rose to 89.17 ± 0.99 % after 5 days of induction (****p < 0.0001 vs. Aβ_40_, one-way ANOVA, Fig. 4d), indicating that Aβ_43_ peptides were mostly insoluble at this stage. However, Aβ_43_ was still significantly more soluble than Aβ_42_ (*p < 0.05, Aβ_43_ vs. Aβ_42_, one-way ANOVA, Fig. 4d).

**Fig. 5:**
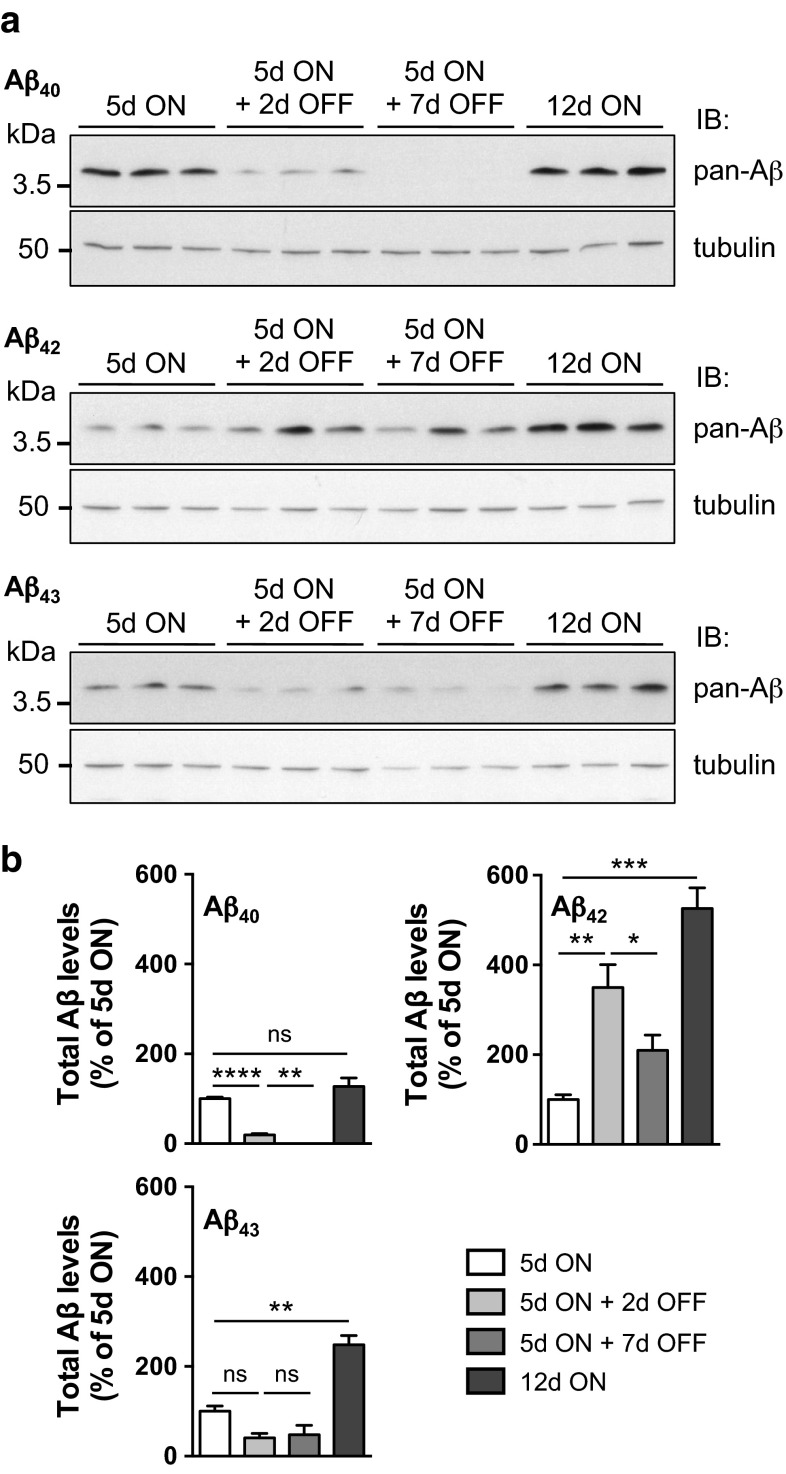
Clearance of Aβ peptides following induction arrest. **a** Western blot analysis of total Aβ amounts retrieved from adult fly head extracts following neuronal (elavGS-driven) expression of Aβ_40_ (*top panel*), Aβ_42_ (*middle panel*) or Aβ_43_ (*lower panel*) following either 5 days of RU486 induction (“5d ON”), 5 days of RU486 induction followed by exposure to RU486-free food for either 2 or 7 days (“5d ON + 2d OFF” and “5d ON + 7d OFF”, respectively), or 12 days of RU486 induction (“12d ON”). Aβ detection was performed using the pan-Aβ 6E10 antibody and Tubulin levels were used for normalization. **b** Quantification of total Aβ levels from **a**. Results were normalized to Tubulin and are expressed relative to levels measured in the “5d ON” condition for each genotype (*p < 0.05, **p < 0.01, ***p < 0.001 and ****p < 0.0001 using one-way ANOVA followed by Tukey’s post hoc test)

Such drastic differences in Aβ solubility might reflect differential ability of cells to clear Aβ peptides. To test this hypothesis, we performed a “switch-on/switch-off” experiment, in which flies were first exposed to the RU486 inducer for a period of 5 days (“5d ON”), to activate neuronal Aβ expression, and then transferred to RU486-free food for either 2 or 7 days (“5d ON + 2d OFF” and “5d ON + 7d OFF”, respectively) to evaluate Aβ clearance either shortly following induction arrest or at a later stage to ensure the complete removal of RU486 from the fly nervous system [26, 28]. A parallel cohort was exposed to the inducer for the entire duration of the experiment, i.e. 12 days (“12d ON”). Using the 6E10 pan-Aβ antibody, we evaluated the total levels of Aβ retrieved from fly head extracts under these conditions (Fig. 5). Interestingly, clearance of Aβ appeared strikingly different depending on the investigated isoform. While Aβ_40_ was rapidly cleared from the fly nervous system, with 19.26 ± 3.03 and 0.36 ± 0.04 % of Aβ_40_ remaining 2 and 7 days following induction arrest, respectively (relative to the levels measured directly following 5 days of induction; ****p < 0.0001, 5d ON + 2d OFF vs. 5d ON and **p < 0.01, 5d ON + 7d OFF vs. 5d ON + 2d OFF, one-way ANOVA, Fig. 5a, b), we could not observe any reduction in Aβ_42_ levels 2 days following induction arrest (Fig. 5a). Rather, Aβ_42_ appeared to accumulate in the fly nervous system, reaching 349.25 ± 50.77 % of the levels observed before the switch to RU486-free conditions (**p < 0.01, 5d ON + 2d OFF vs. 5d ON, one-way ANOVA, Fig. 5b). Seven days following induction arrest, Aβ_42_ levels represented 209.91 ± 33.60 % of the initial “5d ON” levels, suggesting that the clearance process of accumulated Aβ_42_ was effective at this stage (*p < 0.05, 5d ON + 7d OFF vs. 5d ON + 2d OFF, one-way ANOVA, Fig. 5a, b), although the retrieved Aβ_42_ amounts did not reach lower levels than those measured in the “5d ON” condition (*p* > 0.05, 5d ON + 7d OFF vs. 5d ON, one-way ANOVA, Fig. 5b). Interestingly, the behaviour of Aβ_43_ following induction arrest appeared strikingly different from that of both Aβ_40_ and Aβ_42_. Whereas 2 days following induction arrest there was a trend for reduced Aβ_43_ amounts (40.18 ± 9.90 % vs. 5d ON, *p* > 0.05 using one-way ANOVA, Fig. 5a, b), we could not observe any further change in Aβ_43_ levels following 7 days of induction arrest (*p* > 0.05, 5d ON + 7d OFF vs. 5d ON + 2d OFF, one-way ANOVA, Fig. 5a, b). Additionally, the analysis of Aβ_43_ amounts retrieved from head extracts following 12 days of RU486-induced expression revealed a significant Aβ_43_ accumulation in the fly nervous system upon induction (248.09 ± 20.84 % at 12d ON relative to 5d ON levels, **p < 0.01, one-way ANOVA, Fig. 5a, b), while Aβ_40_ expression appeared to yield rather stable total Aβ levels over time (127.27 ± 19.09 % at 12d ON, *p* > 0.05 vs. 5d ON, one-way ANOVA, Fig. 5a, b). In contrast, Aβ_42_ accumulation was striking, reaching after 12 days of induction 525.70 ± 46.10 % of the levels observed following 5 days of induction (***p < 0.001, one-way ANOVA, Fig. 5a, b). Altogether, these results suggest on one hand that Aβ_43_ peptides accumulate in the fly nervous system, though to a lower extent than Aβ_42_, and on the other hand that cells appear to be less efficient in the clearance of Aβ_43_ as compared to that of Aβ_40_. Together with the phenotypic effects of these Aβ isoforms (Figs. 2, 3), these results suggest that Aβ_43_-induced toxicity is related to its high propensity to self-aggregate and accumulate in vivo.

As increasing evidence suggest that toxic Aβ oligomers are important effectors of neurodegeneration (for review, see [2]), we evaluated the presence of PBS-soluble Aβ oligomeric species in our transgenic Aβ fly lines by dot blotting (supplementary Fig. 3a). Immuno-labelling of the membrane with the A11 anti-oligomer antibody did not highlight any specific signal corresponding to soluble oligomers in our transgenic Aβ lines as compared to the elavGS driver control (*p* > 0.05, one-way ANOVA, supplementary Fig. 3a). However, since this antibody is not expected to detect low-molecular weight (MW) Aβ oligomers [16, 18], we performed western blotting on head extracts using the 6E10 pan-Aβ antibody to examine LDS/SDS-stable Aβ assemblies (supplementary Fig. 3b, c). The major LDS/SDS-stable Aβ species for all transgenic lines was monomeric (1-mer, short exposure time, supplementary Fig. 3b), however, longer exposure time revealed the presence of specific 6E10-positive bands of higher MWs, corresponding to the apparent size of Aβ dimers (2-mer, 8 kDa), trimers (3-mer, 12 kDa) and tetramers (4-mer, 16 kDa). Interestingly, the oligomeric profile was different for the investigated Aβ species, with a relative abundance of dimers for the Aβ_42_ line and of trimers for the Aβ_43_ line, while no LDS/SDS-stable low-MW Aβ oligomers could be observed in Aβ_40_-expressing flies (supplementary Fig. 3b, c).

**Fig. 6:**
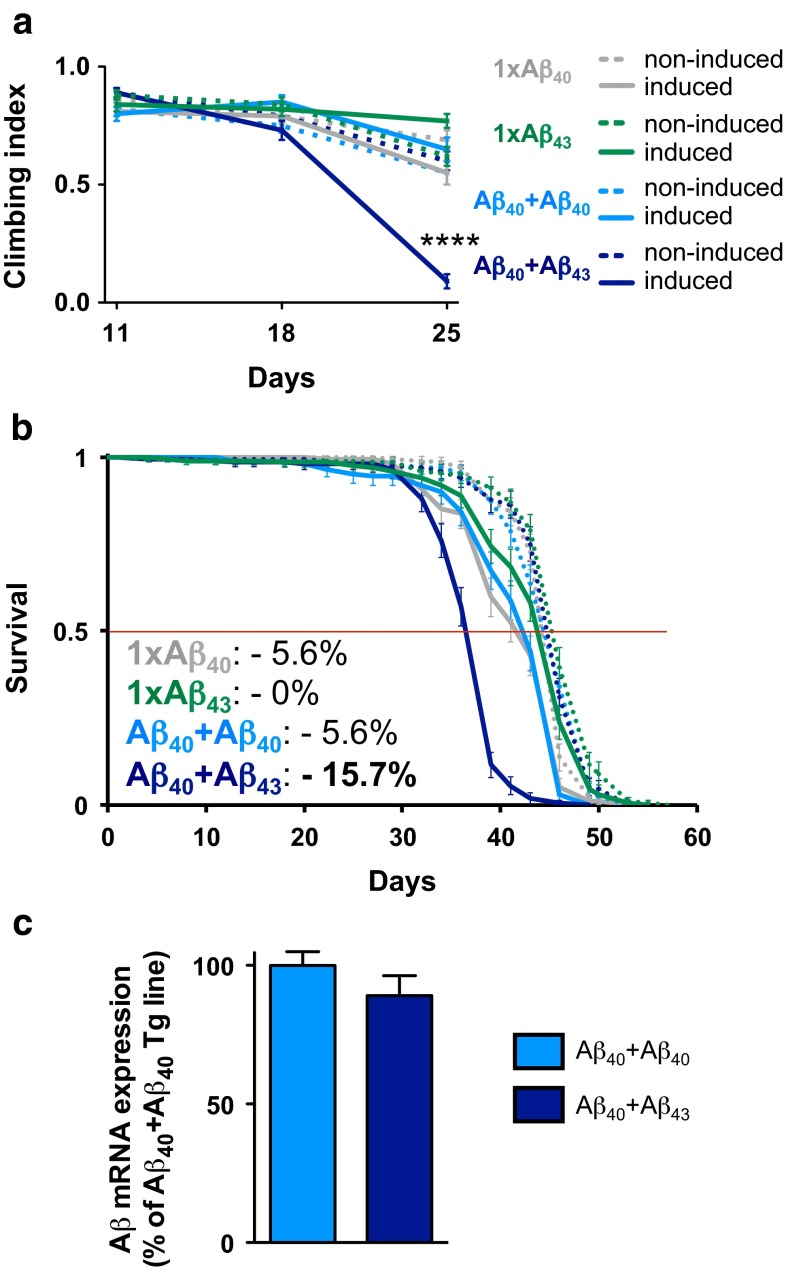
Aβ_43_ triggered toxicity from Aβ_40_. Climbing performance (**a**) and survival curves (**b**) of fly lines expressing 1×Aβ_40_ (*grey*), 1×Aβ_43_ (*green*) or the combination of Aβ_40_+Aβ_40_ (*blue*) or Aβ_40_+Aβ_43_ (*navy blue*) in adult neurons using the elavGS driver. The *inset* shows the percentage of median lifespan reduction vs. non-induced control. ****p < 0.0001 vs. non-induced controls, two-way ANOVA. **c** qRT-PCR analysis of Aβ mRNA levels from head extracts of Aβ_40_+Aβ_40_ and Aβ_40_+Aβ_43_ lines (*p* > 0.05, Student’s *t* test)

Noteworthy, next to its propensity to self-aggregate, Aβ_43_ has also been implicated in the seeding of other Aβ peptides based on in vitro data and co-localisation studies [29, 38], an effect potentially enhancing overall Aβ toxicity. To test whether this process occurred in vivo, we used our *Drosophila* transgenic lines to stoichiometrically induce Aβ_43_ expression together with that of the non-toxic Aβ_40_ and to evaluate whether this combination would trigger toxicity. To that end, we titrated down Aβ_43_ levels by halving transgene copy number from two copies to one, leading to no detectable toxicity in terms of climbing ability (1×Aβ_43_, Fig. 6a) or median lifespan (1×Aβ_43_, Fig. 6b), similar to the effects obtained following halved expression of Aβ_40_ (1×Aβ_40_, Fig. 6a, b). While combining Aβ_40_ with Aβ_40_ itself (Aβ_40_+Aβ_40_) did not lead to any phenotypic changes as compared to Aβ_40_ alone (*p* > 0.05, Fig. 6a, b), we observed that the combination of low levels of both Aβ_40_ and Aβ_43_ induced synergistic toxic effects both on climbing ability (****p < 0.0001 vs. non-induced-Aβ_40_+Aβ_43_ controls and vs. all other induced lines at day 25, two-way ANOVA, Fig. 6a) and survival (median lifespan: −15.7 % of the non-induced controls, *p* < 0.0001, log-rank test, Fig. 6b), suggesting that Aβ_43_ was able to trigger toxicity from Aβ_40_ peptides in vivo, despite the ordinarily harmless nature of the latter. We used the same experimental setup to co-express Aβ_40_ with Aβ_42_ (supplementary Fig. 4), which led to drastic detrimental effects, both on climbing ability (****p < 0.0001 vs. non-induced-Aβ_40_+Aβ_42_ controls and vs. all other induced lines at day 18, two-way ANOVA, supplementary Fig. 4a) and survival (median lifespan: −46.7 % of the non-induced controls, *p* < 0.0001, log-rank test, supplementary Fig. 4b). Even though both Aβ_40_+Aβ_42_ and Aβ_40_+Aβ_43_ combinations led to significant synergistic toxic effects in the fly nervous system, it appeared that the former triggered stronger toxicity. We therefore investigated whether Aβ_43_ could modulate Aβ_42_ toxicity by comparing the effect of Aβ_42_+Aβ_43_ with that of Aβ_42_+Aβ_42_ on climbing ability and survival of flies (supplementary Fig. 5). We could observe a rather modest but yet significant reduction of toxicity in the combined Aβ_42_+Aβ_43_ line both in terms of climbing (**p < 0.01, induced-Aβ_42_+Aβ_43_ vs. induced-Aβ_42_+Aβ_42_, two-way ANOVA, supplementary Fig. 5a) and survival (*p* < 0.0001, induced-Aβ_42_+Aβ_43_ vs. induced-Aβ_42_+Aβ_42_, log-rank test, supplementary Fig. 5b). Importantly, we evaluated Aβ transcript levels among the lines in all three experiments and observed that they were comparable (*p* > 0.05, Aβ_40_+Aβ_40_ vs. Aβ_40_+Aβ_43_, Fig. 6c, Aβ_40_+Aβ_40_ vs. Aβ_40_+Aβ_42_, supplementary Fig. 4c, and Aβ_42_+Aβ_42_ vs. Aβ_42_+Aβ_43_, supplementary Fig. 5c, using Student’s *t* test), implying that the toxic interaction we observed between the Aβ isoforms was taking place at the protein level.

**Fig. 7:**
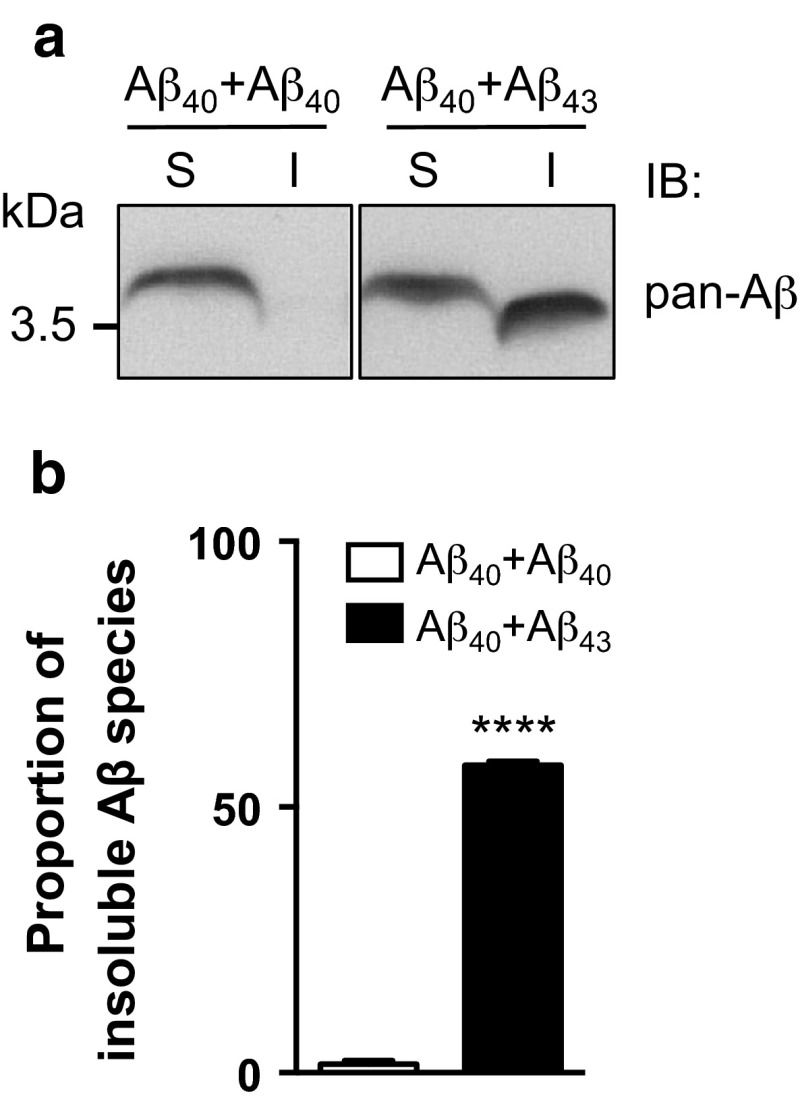
Combining Aβ_40_ with Aβ_43_ enhanced overall Aβ insolubility. **a** Fractionation and western blot of soluble (*S*) and insoluble (*I*) Aβ species retrieved from head extracts of Aβ_40_+Aβ_40_ and Aβ_40_+Aβ_43_ fly lines following induction in adult neurons (elavGS driver), using the pan-Aβ 6E10 antibody. **b** Quantification of the proportion of insoluble Aβ species extracted from Aβ_40_+Aβ_40_ and Aβ_40_+Aβ_43_ fly lines (****p < 0.0001, Student’s *t* test)

**Fig. 8:**
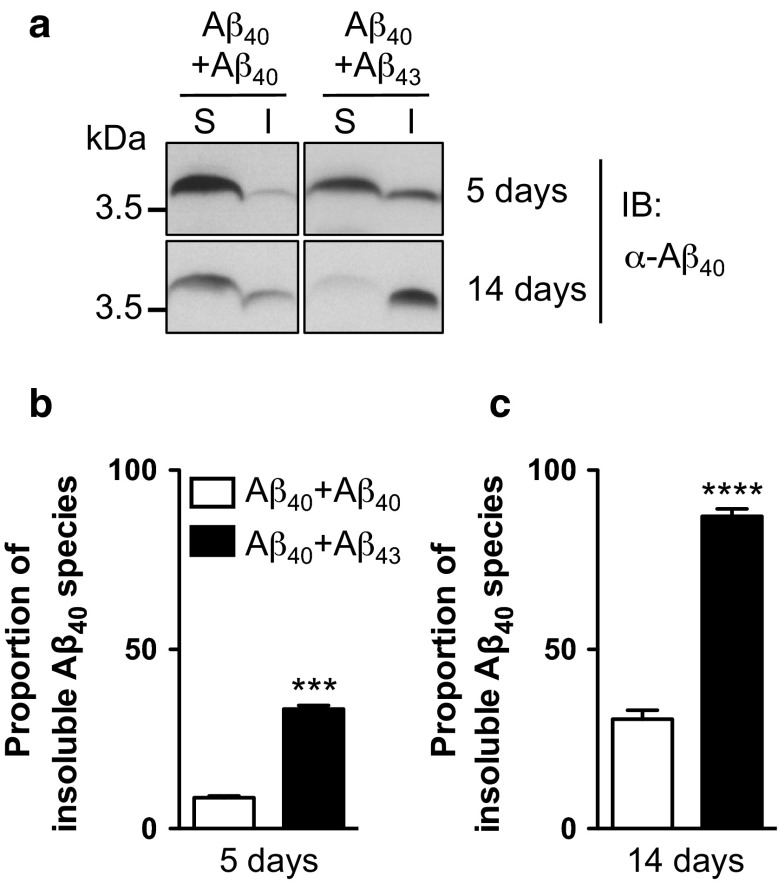
Combining Aβ_40_ with Aβ_43_ enhanced Aβ_40_ insolubility. **a** Western blot analysis of soluble (*S*) and insoluble (*I*) Aβ_40_ species retrieved from head extracts of Aβ_40_+Aβ_40_ and Aβ_40_+Aβ_43_ fly lines after 5 and 14 days of induction in adult neurons (elavGS driver), using an Aβ_40_-specific antibody. Quantification of the proportion of insoluble Aβ_40_ species after 5 (**b**) and 14 (**c**) days of induction (***p < 0.001 and ****p < 0.0001, Student’s *t* test)

**Fig. 9:**
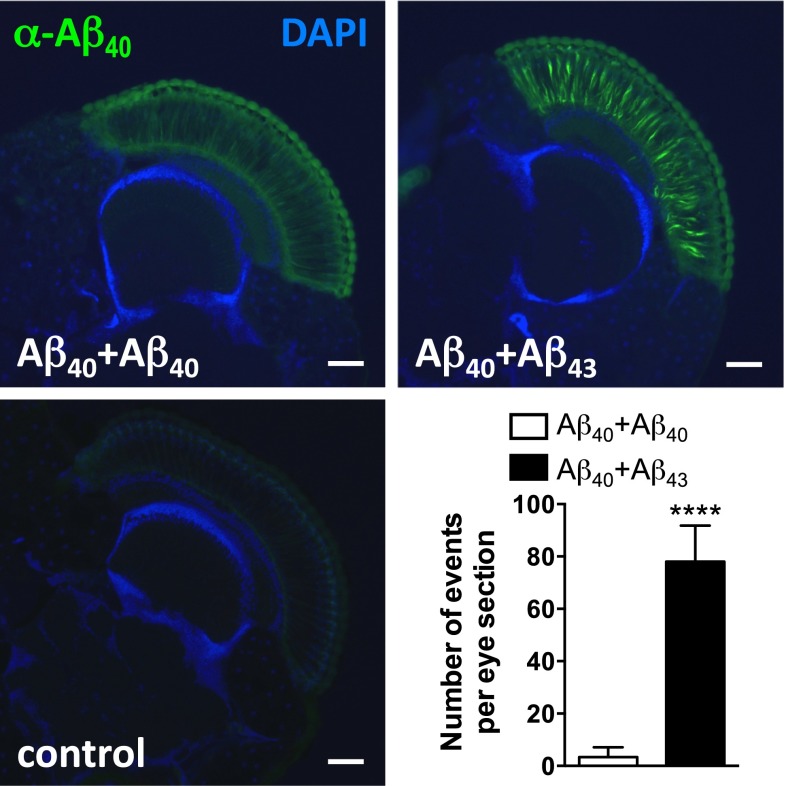
Combining Aβ_40_ with Aβ_43_ enhanced Aβ_40_ immunoreactivity in fly compound eyes. Cryosections from heads of Aβ_40_+Aβ_40_- and Aβ_40_+Aβ_43_-expressing fly lines and of the GMR-Gal4 driver control line were probed with an Aβ_40_-specific antibody (*green*) and counterstained with DAPI (*blue*) and the number of Aβ_40_-positive events per eye section was quantified (****p < 0.0001, Student’s *t* test). *Scale bar* 50 µm

To verify this assumption and to further assess the potential seeding properties of Aβ_43_, we performed fractionation of Aβ according to its solubility in both Aβ_40_+Aβ_43_ and Aβ_40_+Aβ_40_ combined lines. Using the 6E10 pan-Aβ antibody (Fig. 7a), we observed a dramatic decrease of Aβ solubility in the combined Aβ_40_+Aβ_43_ condition as compared to the Aβ_40_+Aβ_40_ line, with insoluble Aβ species representing 57.89 ± 0.79 % of the total Aβ pool in the former line (****p < 0.0001, vs. Aβ_40_+Aβ_40_, Student’s *t* test, Fig. 7a, b). To determine whether this insoluble Aβ pool was holding Aβ_40_ species, which are intrinsically mainly soluble, we analysed the soluble and insoluble protein fractions using an antibody specifically targeting Aβ_40_ species (Fig. 8a). This revealed a progressive shift of Aβ_40_ species towards the insoluble fraction in the Aβ_40_+Aβ_43_ line as compared to the Aβ_40_+Aβ_40_ condition, the former having 33.37 ± 1.04 % of its Aβ_40_ species located in the SDS-insoluble fraction after 5 days of induction (vs. 8.64 ± 0.50 % for the Aβ_40_+Aβ_40_ line, ***p < 0.001, Student’s *t* test, Fig. 8b), while this proportion reached 87.11 ± 2.09 % after 14 days of induction (vs. 30.55 ± 2.49 % for the Aβ_40_+Aβ_40_ line, ****p < 0.0001, Student’s *t* test, Fig. 8c), implying that Aβ_43_ was a potent trigger of Aβ_40_ aggregation in vivo. To strengthen this observation, we performed immunofluorescence experiments on cryosections from *Drosophila* heads following Aβ over-expression in the compound eye, using an antibody specific to Aβ_40_. While the signal appeared predominantly diffuse in the Aβ_40_+Aβ_40_ expressing line, we observed a marked accumulation of Aβ_40_ species in the eye of the Aβ_40_+Aβ_43_ transgenic line (****p < 0.0001, Aβ_40_+Aβ_40_ vs. Aβ_40_+Aβ_43_, Student’s *t* test, Fig. 9), which is in line with the biochemical shift of Aβ_40_ species towards the insoluble protein fraction that we observed in the latter combination (Fig. 8). Altogether, our data indicate that Aβ_43_ species influence Aβ_40_ properties leading to its decreased solubility, which subsequently results in a significant enhancement of toxicity in vivo.

## Discussion

While the amyloid load is built up by a mixture of Aβ peptides in AD brains [2], the respective involvement of these species in AD pathogenesis remains unsolved. Using inducible transgenic *Drosophila* lines expressing human Aβ, we demonstrated in the present study that Aβ_43_ peptides not only self-aggregate and lead to toxicity and neurodegeneration in vivo but also that they can trigger neurotoxicity from the otherwise innocuous Aβ_40_ peptides.

Our transgenic fly lines are based on the direct production of secretory Aβ species. Even though this model bypasses the secretase-based generation of Aβ from its APP precursor, potentially altering the normal trafficking route and sub-cellular localisation of APP and consequently of Aβ, the choice of directly expressing secretory forms of the different Aβ isoforms was crucial to ensure controlled and comparable levels of Aβ peptides produced upon induced expression in the fly nervous system. In addition, by generating transgenic lines using the attP/attB targeted integration system [20], we ensured that insertion of the different Aβ transgenes would take place into the same genomic locus, thereby reducing the risk of a differential promoter regulation due to a different genomic environment. In this way, we ensured that any observed toxic effect would not be a consequence of different transcript levels, which could have otherwise confounded the results, but would instead directly result from differential regulation occurring at the protein level. As an additional control to confirm the comparability of our transgenic lines, we measured the levels of Aβ peptide produced after a very short induction time, i.e. 4 h. We observed no significant difference in Aβ peptide levels between the Aβ transgenic fly lines at this stage, suggesting that translation from Aβ transcripts and therefore Aβ peptide synthesis was occurring at a similar rate, ruling out any potential differences in terms of translation efficiency among the analysed transgenic lines.

Interestingly, however, we observed that the amounts of total Aβ_40_, Aβ_42_ or Aβ_43_ retrieved from *Drosophila* heads became increasingly different with longer induction times. Since transcription and baseline Aβ peptide levels were comparable for all the lines, we hypothesized that such regulation at the peptide level was rather a consequence of altered clearance dynamics. Indeed, by means of a “switch-on/switch-off” experiment, where the RU486 inducer was removed after a period of 5 days, we could observe striking differences regarding the clearance of these Aβ isoforms. Such differential effects are likely to be linked to the unequal propensity of these Aβ species to form SDS-insoluble assemblies, as suggested by the differential kinetics of aggregation displayed by the Aβs. Indeed, among all three isoforms, we could observe that Aβ_42_ was the fastest isoform to generate SDS-insoluble species. Furthermore, even though the kinetics of Aβ_43_ aggregation in the fly nervous system was significantly slower than that of Aβ_42_ as observed after 1 day of induction, it was markedly faster than that of Aβ_40_, which mainly remained in a SDS-soluble state throughout the experiment. As compared to Aβ_40_, Aβ_42_ peptides hold two additional hydrophobic residues that greatly increase their propensity to aggregate [3, 15]. Moreover, recent in vitro studies [3, 15, 29] suggest that Aβ_43_, which bears an additional threonine in its C-terminal compared to Aβ_42_, displays even greater amyloidogenic properties than the latter. Our findings, however, suggest that when directly expressed in the fly nervous system, Aβ_43_ species shift towards an insoluble state with a slower kinetics than Aβ_42_. Although this would require further investigation, one might speculate that the molecular environment found in neuronal cells in vivo influences the kinetics of Aβ aggregation as compared to the behaviour of these peptides in vitro. It is important to note that, even when Aβ_43_ was found in a highly insoluble state in the fly nervous system, i.e. following 5 days of expression, the overall proportion of SDS-insoluble Aβ_43_ species was significantly lower than that of Aβ_42_, suggesting that the additional threonine in Aβ_43_ provides the peptide with an overall higher polarity and therefore lower amyloidogenicity than Aβ_42_. Altogether, our results suggest that Aβ_40_, Aβ_42_ and Aβ_43_ display a differential stability in vivo even when expressed at comparable levels, an effect that is likely related to their unequal propensity to form SDS-insoluble species, eventually influencing the ability of the cells to clear them.

Recent cell culture studies suggested that Aβ_43_ peptides are highly cytotoxic, significantly more so than Aβ_40_ peptides [1, 29]. In line with these data, we measured strong toxic effects resulting from Aβ_43_ expression in either *Drosophila* compound eyes or *Drosophila* adult neurons, as compared to the effects of Aβ_40_. However, we observed that in vivo, Aβ_43_ peptides were significantly less harmful than Aβ_42_ for all measured phenotypes, i.e. eye appearance, climbing ability, survival and neurodegeneration. Among the few cell culture studies that have so far investigated Aβ_43_, the relative cytotoxicity of the latter as compared to Aβ_42_ is still a matter of debate, some studies highlighting a higher toxicity of Aβ_43_ [23, 29], others suggesting that Aβ_42_ is more toxic [1]. Our in vivo results support the latter hypothesis, and it appears that in our system the observed differential toxic effects correlate with the unequal propensity of the Aβs to aggregate and therefore to be cleared from the cells, in agreement with several studies in *Drosophila* showing that Aβ toxicity is correlated with its propensity to form insoluble aggregates [11, 19, 34].

Nevertheless, given the increasing amount of data suggesting that soluble Aβ oligomers are important drivers of neurotoxicity in AD and AD models (for review, see [2]), together with recent data suggesting that Aβ_43_ assembles into soluble oligomers in vitro [31], we further analysed the potential occurrence of Aβ oligomers in our transgenic lines using the A11 oligomer-specific antibody. However, we could not highlight any PBS-soluble A11-positive oligomers in our lines, in agreement with previous studies analysing Aβ_42_ transgenic *Drosophila* [11]. However, since the A11 antibody does not detect low-MW Aβ oligomers, we used the pan-Aβ 6E10 antibody on western blot [18, 22] and could observe the presence of LDS/SDS-stable Aβ assemblies at the apparent MW of dimers, trimers and tetramers, the relative abundance of which differed among the investigated Aβ lines. While our results highlight the relative abundance of LDS/SDS-stable Aβ_43_ trimers and confirm published data that Aβ_42_ can form dimers in *Drosophila* [6, 11], further investigation will be required to decipher the potential contribution of these LDS/SDS-stable Aβ assemblies to the observed phenotypes.

Another important question in the context of AD, where several different Aβ species are found in brain deposits, is whether Aβ_43_ peptides could exacerbate neurotoxicity from other Aβ species in vivo. Therefore, we investigated whether Aβ_43_ could trigger toxicity from the non-toxic Aβ_40_ peptides *in Drosophila*. To that end, we reduced Aβ expression levels to a point where no toxicity could be detected, either on climbing ability or survival, by halving the Aβ transgene copy number from two to one. We combined one copy of each, Aβ_40_ with Aβ_43_ and as a control, Aβ_40_ with Aβ_40_ itself and, importantly, the measured overall Aβ transcript levels were comparable between these combinations. The Aβ_40_+Aβ_43_ combination was significantly more toxic on one hand than Aβ_43_ alone, despite identical Aβ_43_ transgene copy number, and on the other hand than the Aβ_40_+Aβ_40_ combination, even though the overall Aβ transcript levels did not differ between the conditions. At the protein level, the combination of Aβ_40_ with Aβ_43_ increased the proportion of SDS-insoluble Aβ_40_ species as compared to the Aβ_40_+Aβ_40_ line, and shifted the overall Aβ content towards a more insoluble state, suggesting that toxicity arose from an increased propensity to form insoluble structures in the Aβ_40_+Aβ_43_ combined line. Together with published observations that Aβ_43_ peptides can be found in plaque cores in AD brains [29, 36] and that they deposit earlier than other Aβ species in the brain of mouse models of AD [38], our data suggest that Aβ_43_ is involved in the titration of Aβ_40_ and potentially of other Aβ peptides into insoluble structures, therefore acting as a nucleating factor in AD brains. Our results, however, suggest that Aβ_43_ does not further exacerbate toxicity of the already extremely harmful Aβ_42_ in *Drosophila*.

In summary, our findings indicate a remarkable pathogenicity of Aβ_43_ peptides, not only because they self-aggregate and exert potent neurotoxic effects in vivo, but also because of their ability to trigger aggregation and toxicity from other Aβ species. Our results delineate the important contribution of Aβ_43_ peptides to the pathological events leading to neurodegeneration in AD, and suggest that these species represent a relevant target for the treatment of this disease.

## Electronic supplementary material

Supplementary material 1: **Fig.** **1. Quantification of eye phenotypes.** Quantification of the extent of eye roughening induced by the constitutive expression of Aβ_40_, Aβ_42_ or Aβ_43_ in the fly compound eye. Results are expressed as the percentage of analysed flies (moderate phenotype: *p < 0.05: Aβ_43_ vs. Aβ_42_; **p < 0.01: Aβ_43_ vs. Aβ_40_ and Aβ_43_ vs. GMR control; strong phenotype: #p < 0.05, Aβ_43_ vs. Aβ_42_; ##p < 0.01, Aβ_42_ vs. Aβ_40_ and Aβ_42_ vs. GMR control using Fisher’s exact test). (PDF 56 kb)

Supplementary material 2: **Supplementary Fig.** **2. Control experiments for the elavGS driver line.** Climbing ability (**a**) and survival curves (**b**) of the elavGS driver control were analysed (RU486: plain lines, control food: dotted lines). The inset shows the percentage of median lifespan reduction vs. non-induced control. (PDF 65 kb)

Supplementary material 3: **Supplementary Fig.** **3. Characterization of Aβ oligomers. a.** Dot blot analysis (left) of PBS-soluble fractions retrieved from head extracts of 5 days-old Aβ_40_, Aβ_42_ and Aβ_43_ transgenic flies and elavGS controls using the A11 oligomer-specific antibody. Quantification of A11 immunoreactivity is shown on the right panel. **b.** Heads of 14 days-old Aβ_40_, Aβ_42_ and Aβ_43_ transgenic flies and elavGS controls were directly extracted into LDS loading buffer and analysed by western blot using the pan-Aβ 6E10 antibody. Short exposure time (top panel) showed the levels of monomeric Aβ (1-mer) while longer exposure (bottom panel) revealed higher Aβ bands at the apparent size of dimers (2-mer), trimers (3-mer) and tetramers (4-mer) and pointed to a differential oligomeric profile between the transgenic lines. No oligomeric Aβ species could be observed in extracts from the Aβ_40_-expressing flies and no signal was detected in the negative control in this range of molecular weight (elavGS driver line). Western blot for Actin reveals a comparable loading for all the lanes. c. The densitometry profiles show the relative intensity of the Aβ bands for Aβ_42_ and Aβ_43_ transgenics. (PDF 6345 kb)

Supplementary material 4: **Supplementary Fig.** **4. Aβ**_**42**_ **triggered toxicity from Aβ**_**40**_ **. a and b.** Climbing performance (**a**) and survival curves (**b**) of fly lines expressing 1xAβ_40_ (grey), 1xAβ_42_ (violet) or the combination of Aβ_40_+Aβ_40_ (blue) or Aβ_40_+Aβ_42_ (orange) in adult neurons using the elavGS driver. The inset shows the percentage of median lifespan reduction vs. non-induced control. ****p < 0.0001 vs. non-induced controls, two-way ANOVA. **c.** qRT-PCR analysis of Aβ mRNA levels from head extracts of Aβ_40_+Aβ_40_ and Aβ_40_+Aβ_42_ lines (p > 0.05, Student’s *t* test). (PDF 141 kb)

Supplementary material 5: **Supplementary Fig.** **5. Aβ**_**43**_ **did not exacerbate Aβ**_**42**_ **-induced toxicity. a and b.** Climbing performance (**a**) and survival curves (**b**) of fly lines expressing the combination of either Aβ_42_+Aβ_42_ (purple) or Aβ_42_+Aβ_43_ (pink) in adult neurons using the elavGS driver. The inset shows the percentage of median lifespan reduction vs. non-induced control. **p < 0.01, induced-Aβ_42_+Aβ_42_ vs. induced-Aβ_42_+Aβ_43_ at day 11, two-way ANOVA. **c.** qRT-PCR analysis of Aβ mRNA levels from head extracts of Aβ_42_+Aβ_42_ and Aβ_42_+Aβ_43_ lines (p > 0.05, Student’s *t* test). (PDF 133 kb)
