# Supplementary figures and images for: Aβ43 is neurotoxic and primes aggregation of Aβ40 in vivo
Source: Acta Neuropathol. 2015 Apr 11;130(1):35–47. doi: 10.1007/s00401-015-1419-y (PMC4469414; doi:10.1007/s00401-015-1419-y)

Supplementary Figure-1-Partridge. Quantification of eye phenotypes.

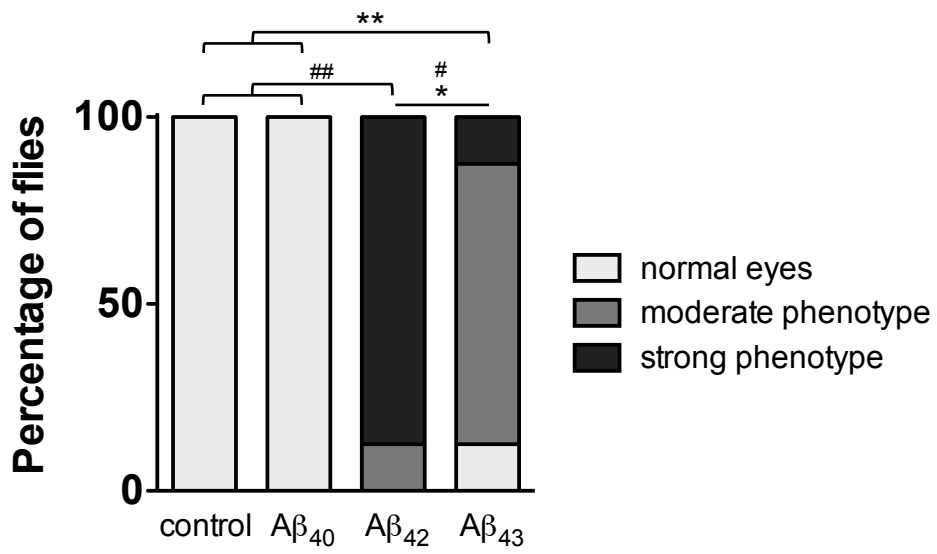

Supplement: Supplementary file 1 — Supplementary material 1: Fig. 1. Quantification of eye phenotypes. Quantification of the extent of eye roughening induced by the constitutive expression of Aβ40, Aβ42 or Aβ43 in the fly compound eye. Results are expressed as the percentage of analysed flies (moderate phenotype: *p < 0.05: Aβ43 vs. Aβ42; **p < 0.01: Aβ43 vs. Aβ40 and Aβ43 vs. GMR control; strong phenotype: #p < 0.05, Aβ43 vs. Aβ42; ##p < 0.01, Aβ42 vs. Aβ40 and Aβ42 vs. GMR control using Fisher’s exact test). (PDF 56 kb) [file 401_2015_1419_MOESM1_ESM.pdf]

Supplementary Figure-2-Partridge. Control experiments for the elavGS driver line.

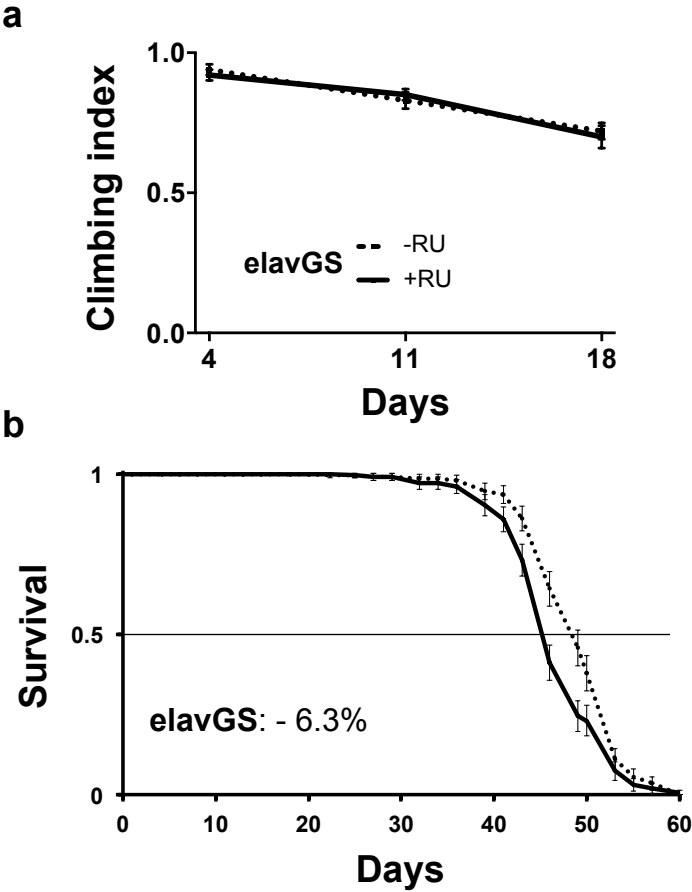

Supplement: Supplementary file 2 — Supplementary material 2: Supplementary Fig. 2. Control experiments for the elavGS driver line. Climbing ability (a) and survival curves (b) of the elavGS driver control were analysed (RU486: plain lines, control food: dotted lines). The inset shows the percentage of median lifespan reduction vs. non-induced control. (PDF 65 kb) [file 401_2015_1419_MOESM2_ESM.pdf]

Supplementary-Figure-3-Partridge. Characterization of Aβ oligomers.

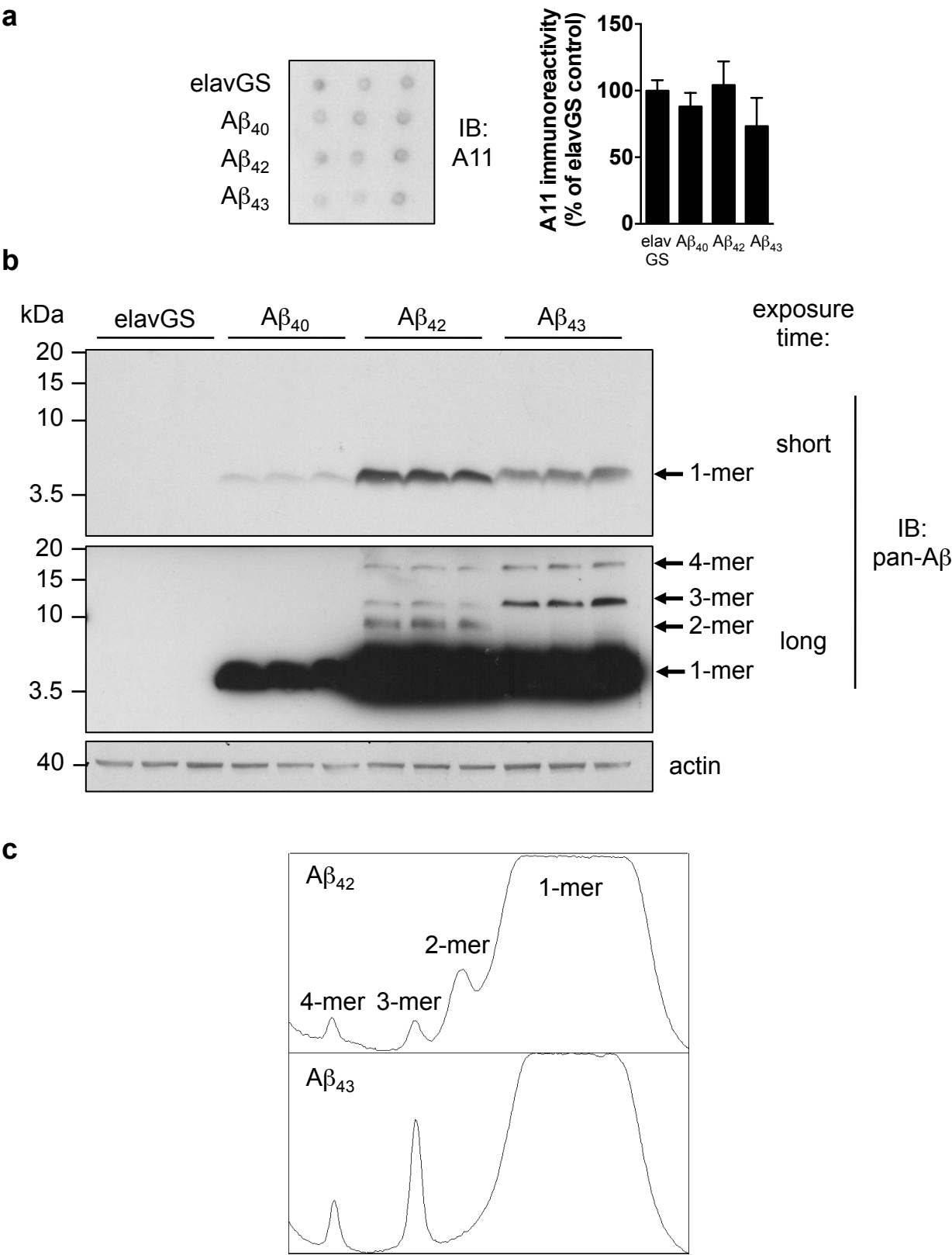

Supplement: Supplementary file 3 — Supplementary material 3: Supplementary Fig. 3. Characterization of Aβ oligomers. a. Dot blot analysis (left) of PBS-soluble fractions retrieved from head extracts of 5 days-old Aβ40, Aβ42 and Aβ43 transgenic flies and elavGS controls using the A11 oligomer-specific antibody. Quantification of A11 immunoreactivity is shown on the right panel. b. Heads of 14 days-old Aβ40, Aβ42 and Aβ43 transgenic flies and elavGS controls were directly extracted into LDS loading buffer and analysed by western blot using the pan-Aβ 6E10 antibody. Short exposure time (top panel) showed the levels of monomeric Aβ (1-mer) while longer exposure (bottom panel) revealed higher Aβ bands at the apparent size of dimers (2-mer), trimers (3-mer) and tetramers (4-mer) and pointed to a differential oligomeric profile between the transgenic lines. No oligomeric Aβ species could be observed in extracts from the Aβ40-expressing flies and no signal was detected in the negative control in this range of molecular weight (elavGS driver line). Western blot for Actin reveals a comparable loading for all the lanes. c. The densitometry profiles show the relative intensity of the Aβ bands for Aβ42 and Aβ43 transgenics. (PDF 6345 kb) [file 401_2015_1419_MOESM3_ESM.pdf]

Supplementary-Figure-4-Partridge. A $\beta_{42}$  triggered toxicity from A $\beta_{40}$

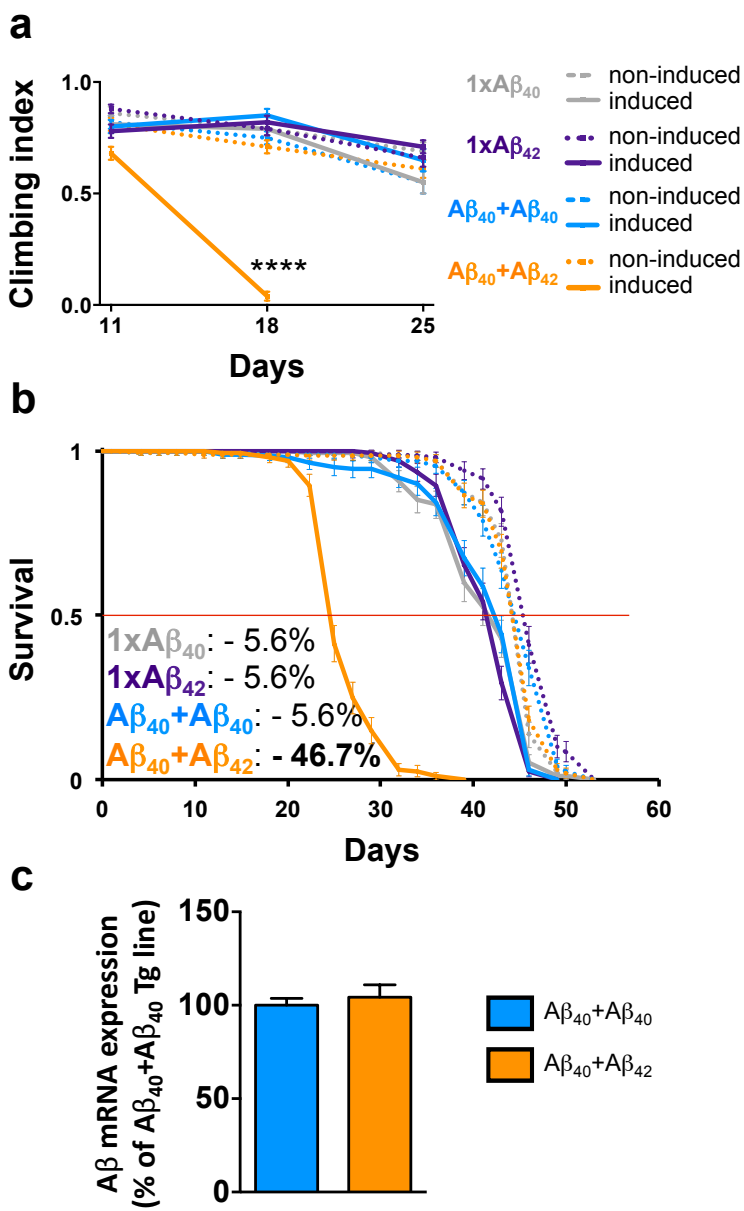

Supplement: Supplementary file 4 — Supplementary material 4: Supplementary Fig. 4. Aβ 42 triggered toxicity from Aβ 40 . a and b. Climbing performance (a) and survival curves (b) of fly lines expressing 1xAβ40 (grey), 1xAβ42 (violet) or the combination of Aβ40+Aβ40 (blue) or Aβ40+Aβ42 (orange) in adult neurons using the elavGS driver. The inset shows the percentage of median lifespan reduction vs. non-induced control. ****p < 0.0001 vs. non-induced controls, two-way ANOVA. c. qRT-PCR analysis of Aβ mRNA levels from head extracts of Aβ40+Aβ40 and Aβ40+Aβ42 lines (p > 0.05, Student’s t test). (PDF 141 kb) [file 401_2015_1419_MOESM4_ESM.pdf]

Supplementary-Figure-5-Partridge.  $A\beta_{43}$  did not exacerbate  $A\beta_{42}$ -induced toxicity.

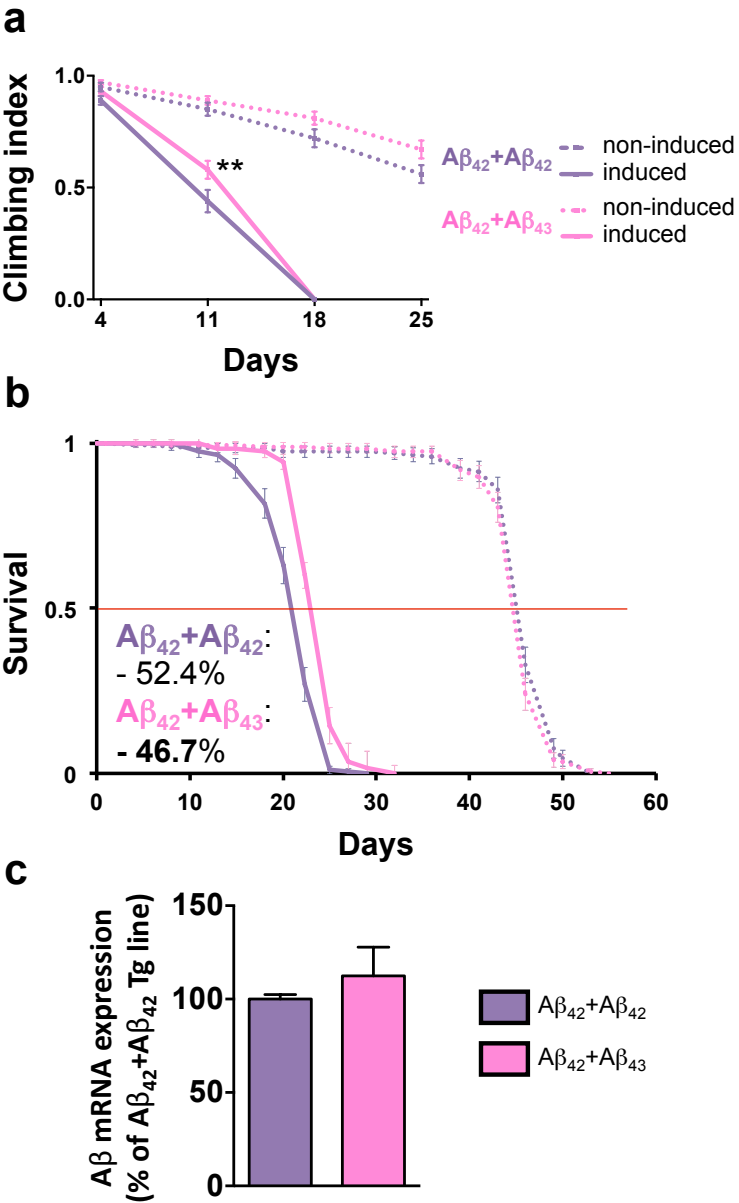

Supplement: Supplementary file 5 — Supplementary material 5: Supplementary Fig. 5. Aβ 43 did not exacerbate Aβ 42 -induced toxicity. a and b. Climbing performance (a) and survival curves (b) of fly lines expressing the combination of either Aβ42+Aβ42 (purple) or Aβ42+Aβ43 (pink) in adult neurons using the elavGS driver. The inset shows the percentage of median lifespan reduction vs. non-induced control. **p < 0.01, induced-Aβ42+Aβ42 vs. induced-Aβ42+Aβ43 at day 11, two-way ANOVA. c. qRT-PCR analysis of Aβ mRNA levels from head extracts of Aβ42+Aβ42 and Aβ42+Aβ43 lines (p > 0.05, Student’s t test). (PDF 133 kb) [file 401_2015_1419_MOESM5_ESM.pdf]
